# Effects of Stretching or Strengthening Exercise on Spinal and Lumbopelvic Posture: A Systematic Review with Meta-Analysis

**DOI:** 10.1186/s40798-024-00733-5

**Published:** 2024-06-05

**Authors:** Konstantin Warneke, Lars Hubertus Lohmann, Jan Wilke

**Affiliations:** 1 Institute of Sport Science, Department of Movement Sciences, Alpen-Adrian-University Klagenfurt, Klagenfurt, Austria; 2 Department of Human Movement Science and Exercise Physiology, Institute of Sport Science, Friedrich Schiller University, Jena, Germany

**Keywords:** Muscular Imbalance, Stretching, Strengthening, Back pain, Forward head Posture, Pelvic tilt, Forward Shoulder, Kyphosis, Lordosis

## Abstract

**Background:**

Abnormal posture (e.g. loss of lordosis) has been associated with the occurrence of musculoskeletal pain. Stretching tight muscles while strengthening the antagonists represents the most common method to treat the assumed muscle imbalance. However, despite its high popularity, there is no quantitative synthesis of the available evidence examining the effectiveness of the stretch-and-strengthen approach.

**Methods:**

A systematic review with meta-analysis was conducted, searching PubMed, Web of Science and Google Scholar. We included controlled clinical trials investigating the effects of stretching or strengthening on spinal and lumbopelvic posture (e.g., pelvic tilt, lumbar lordosis, thoracic kyphosis, head tilt) in healthy individuals. Effect sizes were pooled using robust variance estimation. To rate the certainty about the evidence, the GRADE approach was applied.

**Results:**

A total of 23 studies with 969 participants were identified. Neither acute (d = 0.01, *p* = 0.97) nor chronic stretching (d=-0.19, *p* = 0.16) had an impact on posture. Chronic strengthening was associated with large improvements (d=-0.83, *p* = 0.01), but no study examined acute effects. Strengthening was superior (d = 0.81, *p* = 0.004) to stretching. Sub-analyses found strengthening to be effective in the thoracic and cervical spine (d=-1.04, *p* = 0.005) but not in the lumbar and lumbopelvic region (d=-0.23, *p* = 0.25). Stretching was ineffective in all locations (*p* > 0.05).

**Conclusion:**

Moderate-certainty evidence does not support the use of stretching as a treatment of muscle imbalance. In contrast, therapists should focus on strengthening programs targeting weakened muscles.

**Supplementary Information:**

The online version contains supplementary material available at 10.1186/s40798-024-00733-5.

## Key Points

• Stretching of tight muscles and strengthening of weak muscles is popular in treating muscular imbalance of the pelvis and spine. While combined interventions have previously been meta-analyzed and shown to be effective, the effectiveness of both used in isolation has not been investigated.

• This meta-analysis found no effects of stretching on posture while strengthening can improve imbalances/posture.

• Additional studies including higher stretching volumes and intensities are warranted.

**Supplementary Information:**

The online version contains supplementary material available at 10.1186/s40798-024-00733-5.

## Background

Spinal alignment and posture have been investigated for about 250 years [1, 2]. Evidence syntheses from recent decades suggest that deviations from the assumed physiological norm may be associated with the occurrence of musculoskeletal pain. Chun et al. [3] found a strong cross-sectional relationship of reduced lumbar lordosis and low back pain. In a meta-analysis of prospective cohort studies, limited lordosis predicted the development of low back pain with an odds ratio of 1.27 [4]. With regard to the neck, patients with pain displayed a forward head posture (FHP) when compared to asymptomatic individuals. Interestingly, the magnitude of FHP correlated with neck pain intensity and subjective disability [5], which is frequently associated with, for instance, early fatigue, neck and shoulder pain, decreased respiratory capacity, as well as reduced aerobic endurance [6, 7]. Barrett et al. [5] focused on thoracic kyphosis. The authors found that persons with excessive spinal curvature exhibited reductions in shoulder range of motion. This is of relevance because restricted shoulder mobility has been shown to increase the risk for upper extremity pain and injury [8, 9].

Changes of lumbopelvic or spinal posture are commonly related to muscle imbalance [10]. Such imbalance is suggested to originate from extended periods of biomechanical, psychological and social stresses as well as repetitive activities [11, 12] While some muscles respond with tightness or shortening, their antagonists may become too weak to maintain the normal joint position [13–18]. As an example for muscle imbalance, Janda [13, 14] hypothesized that shortening of the pectoralis major, upper trapezius and levator scapulae muscles in conjunction with weakness of the deep neck flexors, lower trapezius and rhomboids causes excessive kyphosis and FHP.

Besides various other methods including mobilization [19, 20], yoga [21], Pilates [22, 23], manual therapy [24], or taping [25], stretching of tight muscles and strengthening of weak muscles has gained high popularity in the treatment of muscle imbalance. A survey by Perriman and colleagues from 2012 [26] revealed that 71% and 64% of the physiotherapists use stretching and strengthening, respectively, to treat excessive kyphosis, while in 2024, 60% of the physiotherapists and sport scientists attending an Austrian training convention assumed stretching to be effective in treating muscular imbalance [27]. Despite the frequent use of the stretch-and-strengthen approach, the effectiveness of corrective exercise routines on posture is questionable [15, 16]. A systematic review with meta-analysis by Gonzalez-Galvez et al. [18] reported a positive influence of exercise programs in general, mostly when combining stretch and strengthening exercise. Interestingly, they concluded that strengthening may be superior to stretching. Yet, this assumption was based on the analysis of only 10 studies and, more importantly, no investigation of the isolated effects of stretching and stretching was performed. Withers et al. [28] included different training approaches. Among these, they examined stretching as a stand-alone treatment for hyperkyphosis. Since only one isolated static stretching was found, further research seems necessary. In view of the lack of evidence on the individual components of the stretch-and-strengthen approach, the present systematic review with meta-analysis was conducted to summarize the evidence on isolated stretch and strengthening treatments aiming to modify spinal or lumbopelvic posture.

## Methods

A systematic review with meta-analysis was performed adhering to the PRISMA (Preferred Reporting Items for Systematic Reviews and Meta-Analyses) guidelines. We considered ethical publishing standards [29] and registered the study in the PROSPERO database (CRD42023412854).

### Literature Search

**Fig. 1:**
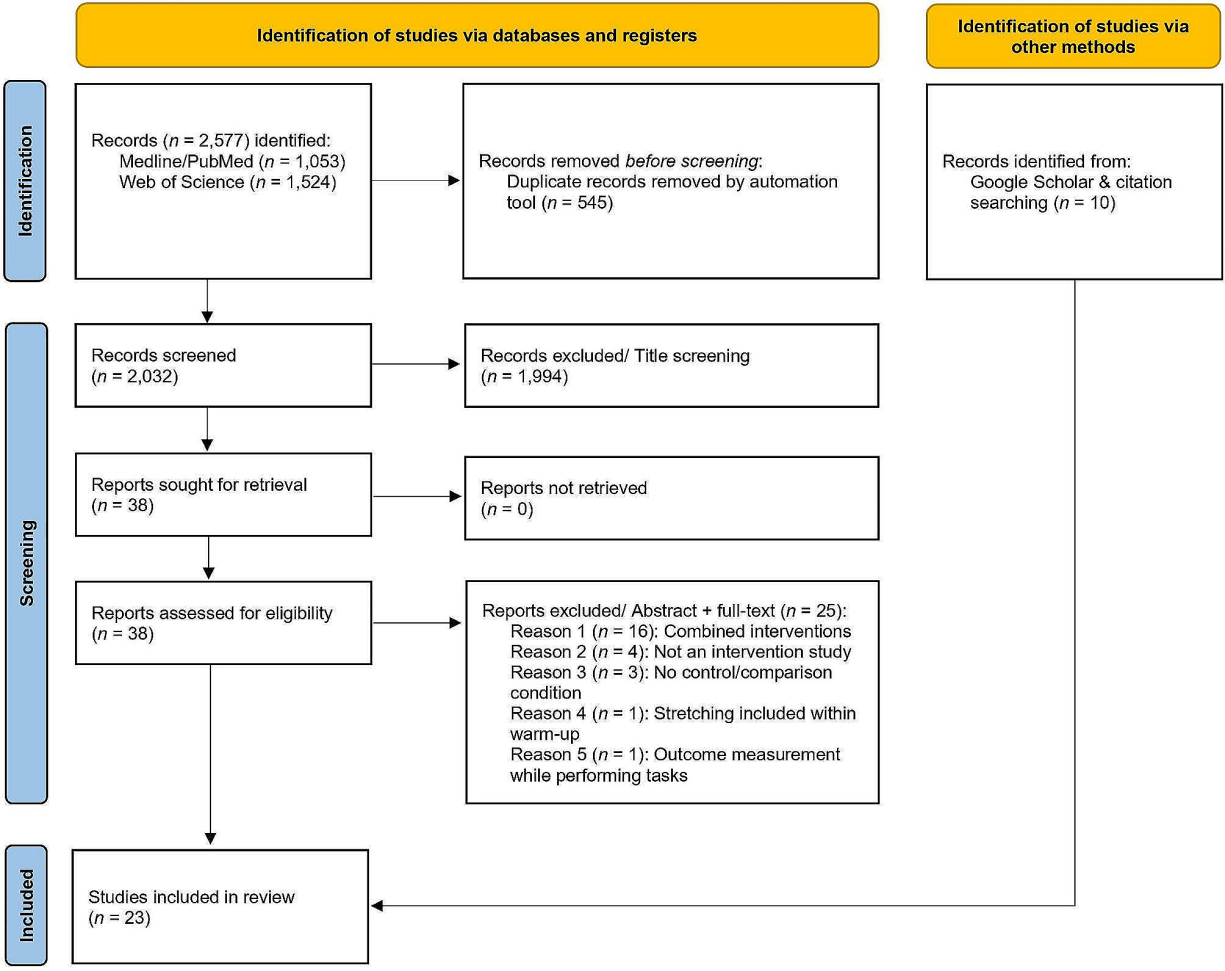
Flow-chart of literature search for studies assessing the influence of stretching or strengthening on posture

Two authors (KW & LHL) conducted a systematic literature search using MEDLINE/PubMed and Web of Science (inception to April, 2023) and assessed all records independently. Disagreements at each screening level (title, abstract + full-text) were resolved by discussion (see Fig. 1). Database queries were supplemented by a hand search using Google Scholar as well as citation searching in eligible studies. The following criteria were applied for study inclusion: (1) randomized or non-randomized controlled intervention study design, (2) assessment of acute (post-testing immediately following the intervention) or chronic (intervention period of at least one week) effects, (3) comparison of stretching vs. strengthening, stretching vs. non-intervention control, or strengthening vs. non-intervention control, (4) measurement of pelvic tilt, lumbar lordosis, kyphosis, and/or forward head/forward shoulder posture using objective and quantifiable measurements (e.g., radiographs or camera systems), (5) inclusion of healthy adults. Patients with a history of musculoskeletal, neurologic, or cardiopulmonary disorders, joint replacements, osteoporosis, specific back pain or other pathologies were excluded from this analysis to improve homogeneity. Trials combining different interventions (i.e., stretching plus strengthening) were excluded as well.

Stretching interventions eligible for inclusion were static, dynamic and ballistic stretching and proprioceptive neuromuscular facilitation in accordance with Warneke & Lohmann [30] and Behm [31]. Static stretching was defined as muscle lengthening until onset of a stretch sensation or to the point of discomfort. By definition, this position is to be held and can be performed passively via partner, external weight or a tool, or actively via movement. Proprioceptive neuromuscular facilitation includes a (sub-) maximal voluntary contraction to a stretching bout with or without antagonist contraction. Dynamic stretching was defined as controlled back-and-forth movement in the end range of motion with ballistic stretching as a sub-category and less controlled, bouncing movements [32]. Strengthening interventions were considered eligible if the authors stated the application of dynamic or isometric muscle actions sufficient to increase strength capacity, while the control group was considered to be inactive if no structured intervention was performed within the study.

The search terms were created based on the requirements of each database (see Appendix S1). In addition to the database searches, the reference lists of all included studies were screened for further eligible articles [33].

### Methodological Study Quality and Risk of Bias

We used the PEDro scale for the assessment of methodological study quality [34, 35]. Scoring was performed by two independent investigators (KW & LHL). If both did not reach consensus, a third examiner provided the decisive vote (JW) [28]. To estimate the risk of publication bias, funnel plots, created using the modification of Fernandez-Castilla et al. [36] for multiple study outcomes, were visually inspected. In addition, we performed Egger’s regression test with the extension for dependent effect sizes [36].

To rate the certainty about the evidence, we applied the GRADE working group criteria [37]. Briefly, the quality of evidence of randomized, controlled trials was initially classified as high and adjusted afterwards, considering the GRADE framework. In detail, in case of limitations in study design or execution, inconsistency of results, indirectness of evidence, imprecision or publication bias, one point was subtracted for each weakness. On the contrary, large magnitude effects or a dose-response gradient led to improvements of the quality of evidence by one point each. This resulted in a final rating of the certainty about the evidence as very low, low, moderate, or high.

### Data Processing and Statistics

The means (M) and standard deviations (SD) from pre- and post-tests were extracted for all parameters (e.g. lordotic angle). In case of missing data, the authors of the primary studies were contacted. KW and LHL extracted data from eligible studies cooperatively, meaning that one read the values aloud and checked the shared screen while the other entered the numbers in a Microsoft Excel sheet. Additionally, KW double-checked the entered values for accuracy at the end of the extraction process. Changes from pre- to post-test were calculated as M_(posttest)_ – M_(pretest)_ and standard deviations were pooled as

$$S{D_{pooled}} = \sqrt {\frac{{\left( {{n_1} - 1} \right)*SD_1^2 + \left( {{n_2} - 1} \right)*SD_2^2}}{{\left( {{n_1} - 1} \right) + ({n_2} - 1)}}} .$$

A meta-analysis with robust variance estimation, accounting for the dependency of effect sizes (e.g. in case of multiple outcomes in the same study), was performed to pool the standardized mean differences (SMD) and 95% confidence intervals (CI) between the intervention (stretching or strengthening) and control groups [38]. The between-study variance component was estimated using τ^2^. Pooled effect sizes (ES) were interpreted as follows: 0 ≤ ES < 0.2 trivial, 0.2 ≤ ES < 0.5 small, 0.5 ≤ ES < 0.8 moderate and ES ≥ 0.8 large [39]. Besides the omnibus analyses on the effects of stretching and strengthening, we performed sub-analyses for different body regions (1: forward head posture/thoracic kyphosis, 2: pelvic angle/lordotic angle). All calculations were performed using R and the robumeta package [40].

## Results

### Search Results and Study Characteristics

Figure 1 shows the flow-chart of the literature search.

A total of 23 studies [41–63] (*n* = 969 participants, 48 ES) were found eligible. Fourteen of the papers examined the effects of stretching [41, 43, 45, 46, 51–55, 57–59, 61, 62] while fifteen studies [42–44, 46–52, 56, 59, 60, 62, 63] investigated the effects of strengthening. The majority of the studies (*n* = 21) focused on chronic treatment effects while only 2 studies explored acute effects. These were quantified via the Cobb angle, kyphosis angle, lordosis angle, head tilt angle, neck flexion angle, hip extension angle, acromion process vertical distance and assessed with marker-based camera (three-dimensional) motion capture systems, radiography, the spinal mouse system, steel ruler, photographs, flexible rulers, inclinometers and goniometers. Most studies (*n* = 17) included participants without pain. While patients were generally excluded, six studies included participants with unspecific back (*n* = 2) [54, 59] or neck (*n* = 4) [51, 52, 60, 63] pain. Table 1 provides information about the studies’ characteristics.

**Table 1:** Characteristics of included studies

Study	Participants	Stretching	Strengthening	Outcome
Fani et al. [41]	*n* = 52 pain-free individuals, SS: 13, MB:13, SS + MB:13, CG:13 Participants with rounded shoulder posture	1wk, 5x/wk 10 × 15 s Pectoralis minor	-	Shoulder position, internal/ upward rotation, anterior tipping, protraction, sitting/walking, 3D-MoCap
Fukuda et al. [42]	*n* = 26 pain-free individuals, IG:13, CG:13 No information on postural abnormalities	-	6 months, at least 1x/wk 10reps/set, 20–30 min Back extensors	Thoracic kyphosis, lumbar lordosis, sacral inclination, head posture during standing via spinal mouse system and camera system
Hajihosseini et al. [43]	*n* = 40 pain-free individuals SS:10, STR:10, CB:10, CG:10 Participants with forward shoulder posture	6 weeks, 3/week 6-12 × 10–15 s Pectoralis minor	6 wks, 3/wk 3 × 10-20 reps Trapezius, rhomboideus	Forward shoulder angle, standing photographs with 3 anatomical landmarks
Hamidiyeh et al. [44]	*n* = 24 pain-free individuals, IG:12, CG:12 Participants with excessive thoracic kyphosis	-	8 wks, 3x/wk 60 min per day	Kyphosis angle measurement using a flexible ruler, no further information
Hammonds et al. [45]	*n* = 34 pain-free individuals, IG: 18, CG:16 No information on postural abnormalities	Acute 3 × 30 s Hamstrings	-	Pelvic tilt with maximum hip flexion and maximum knee extension in running movements via MoCap system
Hassan et al. [46]	*n* = 34 pain-free individuals, IG:17, CG:17 Participants with forward head/shoulder posture	10 weeks, 3x/week - sec Shoulder muscles	10 wks, 3x/wk 1 × 5 repetition for shoulder muscles	Craniovertebral angle and shoulder angle in standing position measured via marker-based camera system
Im et al. [63]	*n* = 15 individuals with neck pain, IG:8, CG:7 Participants with forward head posture	-	4 wks,3x/wk 30 min of training Scapular stabilization	Cervical angle measurement, no further information
Itoi & Sinaki [47]	*n* = 60 pain-free individuals, IG:32, CG:28 Participants with estrogen-deficiency	-	2 years, 5x/wk 10 repetitions Back extensors	Thoracic kyphosis, lumbar lordosis and sacral inclination in a standing position via lateral roentgenogram evaluation
Katzman et al. [49]	*n* = 99 pain-free individuals, IG: 51, CG:48 Participants with excessive thoracic kyphosis	-	24 wks, 3x/wk 1 h of training	Cobb kyphosis angle in a standing position via lateral spine radiographs
Katzman et al. [48]	*n* = 103 pain-free individuals, IG: 54, CG:49 Participants with excessive thoracic kyphosis	-	12 wks, 2x/week 1 h of training	Cobb kyphosis angle in a standing position via lateral spine radiographs
Kim et al. [50]	*n* = 30 pain-free individuals, IG:15, CG: 15 Participants with forward head posture	-	4 wks, 3x/wk 15-20reps, 7 exercises	Craniovertebral angle in a standing position via 2D camera system
Lee & Lee [51]	*n* = 21 individuals with neck pain, SS:10, STR:11 Participants with forward head posture	2 weeks, 3x/week 3 × 15–20 s Shoulder muscles	2 wks, 3x/wk 3 × 10 reps Shoulder muscles	Forward head angle measurement in a standing position via 2D camera system using 3 landmarks
Lee et al. [52]	*n* = 30, individuals with neck pain, SS:15, STR:15 No information on postural abnormalities	8 weeks, 5/week 30 min stretching program Shoulder muscles	8 wks, 5/wk Isometric training program 10 repetitions, 10 s	Head tilt angle, neck flexion angle, forward shoulder angle and craniocervical flexion in a standing position via X-ray photographs
Li et al. [53]	*n* = 39 pain-free individuals, IG: 19, CG:20 Participants with tight hamstrings, no information on postural abnormalities	3 wks, daily 10 × 15 s hamstrings	-	Lumbar & hip extension angle measurement in a full forward bent position and partial bent position using an electromechanical digitizer
Malai et al. [54]	*n* = 20 individuals with non-specific back pain, IG:10, CG:10 No information on postural abnormalities	Acute 5 × 10 s hold relax Hamstrings	-	Lumbar lordosis angle measurement in a lying position (modified Thomas test) using flexible ruler and goniometer
Muyor et al. [55]	*n* = 58 pain-free individuals, IG:27, CG: 31 No info on postural abnormalities	12 weeks, 3x/week 3 × 20 s Hamstrings	-	Thoracic and lumbar spine curvature measured in a lying and sitting position (SLR, toe touch test) using electromechanical spinal mouse system (C7 to S3)
Nitayarak & Charntaraviroj [56]	*n* = 39 pain-free individuals, IG:19, CG:20 Participants with upper-crossed syndrome	-	4 wks,3x/wk 3 × 10 reps (isometric holds) Trapezius, lower serratus	Cervical angle, right and left shoulder angle, and midthoracic curve were measured in a standing position using a flexi ruler and a marker based 2D camera system
Roddey et al. [57]	*n* = 40 pain-free individuals, IG:25, CG:15 Participants with forward head/shoulder posture	2 weeks, 7x/week 3 × 30 s Pectoralis major	-	Forward shoulder position measurement in a standing position using the total scapular distance, evaluated via the distance between the scapular, measured via diVeta technique using a tape measurement
Rossa et al. [58]	*n* = 28 pain-free individuals, IG:14, CG:14 Participants with tight hamstrings, no information on postural abnormalities	4 weeks, 3x/week 4 × 30 s Hamstring	-	Pelvic tilt angle and lumbar lordosis angle measurements using an inclinometer
Shamsi et al. [59]	*n* = 45 individuals with chronic non-specific back pain, SS:15, STR: 15, CG:15 Participants with tight hamstrings, no information on postural abnormalities	4 weeks, 3x/week Duration: - Hamstrings	4 wks, 3/wk Details: - Hamstrings	Pelvic tilt angle in a standing position using an inclinometer
Sikka et al. [60]	*n* = 30 individuals with neck pain, IG: 15, CG: 15 Participants with tight hamstrings, no information on postural abnormalities	-	4 wks,4x/wk 3 × 10reps, 10 s hold	Craniovertebral angle in a sitting position using a 2D marker-based camera system
Watt et al. [61]	*n* = 82 pain-free individuals, IG:43, CG: 39 Participants with tight hamstrings, no information on postural abnormalities	10 weeks, 7x/week 2 × 2 min per leg Hip flexors	-	Peak hip extension and peak anterior tilt in a walking movement (dynamic gait measurement) using a 10 camera MoCap system, hip extension measurement was performed in a lying position
Yoo [62]	*n* = 20 pain-free individuals, SS:10 STR:10 Participants with forward shoulder posture	2 weeks, frequency: - 3 × 30 s Pectoralis major / minor	2 wks, 6x/wk 2 × 10 repetitions Reverse butterfly, 60–80% 1RM	Forward shoulder angle measured via steel ruler (vertical distance between right acromion process and floor)

SLR = Straight leg raise, SS = Static Stretching, STR = Strengthening, MB = Mobilization, CG = Control Group, CB = combined, - = not applicable, wk = week, wks = weeks, reps = repetitions, min = minutes, s = seconds, IGh = intervention group home training, IGs = intervention group supervised, MoCap = motion capturing system

### Methodological Quality, Risk of Bias and Certainty About the Evidence

For stretching studies, the average risk of bias was rated as fair with a PEDro score of 4.1 ± 1.3 (range: 3 to 8 points). The same applied to strengthening studies, which averaged 4.3 ± 1.4 points (range: 2 to 7). Almost all studies used random group allocation, reported statistical between-group comparisons and provided both, point measures and measures of variability. In contrast, blinding of the participants was only reported in one study, and not at all for therapist blinding. Also, very few studies (*n* = 2) declared application of the intention-to-treat principle (see Table 2).

**Table 2:** Quality assessment using the PEDro scale

Study	2.	3.	4.	5.	6.	7.	8.	9.	10.	11.	Score
Fani et al. [41]	Y	N	Y	Y	N	Y	Y	Y	Y	Y	8/10
Fukuda et al. [42]	Y	N	N	N	N	N	N	N	Y	Y	3/10
Hajihosseini et al. [43]	Y	N	Y	N	N	N	N	N	Y	Y	4/10
Hamidiyeh et al. [44]	Y	N	N	N	N	Y	N	N	Y	Y	4/10
Hammonds et al. [45]	Y	N	N	N	N	N	N	N	Y	Y	3/10
Hassan et al. [46]	Y	N	Y	N	N	N	Y	N	Y	Y	4/10
Im et al. [63]	N	N	N	N	N	N	N	N	Y	Y	2/10
Itoi & Sinaki [47]	Y	N	N	N	N	N	N	N	Y	Y	3/10
Katzman et al. [48]	Y	Y	Y	N	N	Y	Y	N	Y	Y	7/10
Katzman et al. [49]	Y	Y	Y	N	N	Y	Y	N	Y	Y	7/10
Kim et al. [50]	Y	N	Y	N	N	N	N	N	Y	Y	4/10
Lee & Lee [51]	Y	N	Y	N	N	N	N	N	Y	Y	4/10
Lee et al. [52]	Y	N	Y	N	N	N	N	N	Y	Y	4/10
Li et al. [53]	Y	N	N	N	N	N	N	N	Y	Y	3/10
Malai et al. [54]	N	N	N	N	N	Y	N	N	Y	Y	3/10
Muyor et al. [55]	Y	N	Y	N	N	N	N	N	Y	Y	4/10
Nitayarak & Charntaraviroj [56]	Y	Y	N	N	N	Y	Y	N	Y	Y	6/10
Roddey et al. [57]	N	N	Y	N	N	N	Y	N	Y	Y	4/10
Rossa et al. [58]	N	N	Y	N	N	N	Y	Y	Y	Y	5/10
Shamsi et al. [59]	Y	N	Y	N	N	N	N	N	Y	Y	4/10
Sikka et al. [60]	Y	N	Y	N	N	Y	N	N	Y	Y	5/10
Watt et al. [61]	Y	N	N	N	N	Y	N	N	Y	Y	4/10
Yoo [62]	N	N	Y	N	N	N	N	N	Y	Y	3/10

N = No, Y = Yes

Visual inspection of funnel plots suggested absence of a publication bias (Figures A-C in Supplemental material). These results were confirmed by Egger’s regression tests (t = 2.26, *p* = 0.16, 95% CI -0.32–0.99) for chronic stretching, (t=-0.88, *p* = 0.206, 95% CI -2.40–0.64), strengthening, and (t = 0.76, *p* = 0.532, 95% CI -2.06–2.84) chronic stretching vs. strengthening.

With regard to the stretching studies, the certainty about the evidence was downgraded by 1 level (high to moderate) due to (1) risk of bias classified as fair via the PEDro score. For the strengthening studies, due to (1) risk of bias and (2) heterogeneity, certainty was downgraded by 2 levels (high to low) but upgraded one level due to the large effect size. Therefore, in sum, for both stretching and strengthening, the certainty about the evidence was moderate.

### Quantitative Synthesis

#### Stretching

**Fig. 2:**
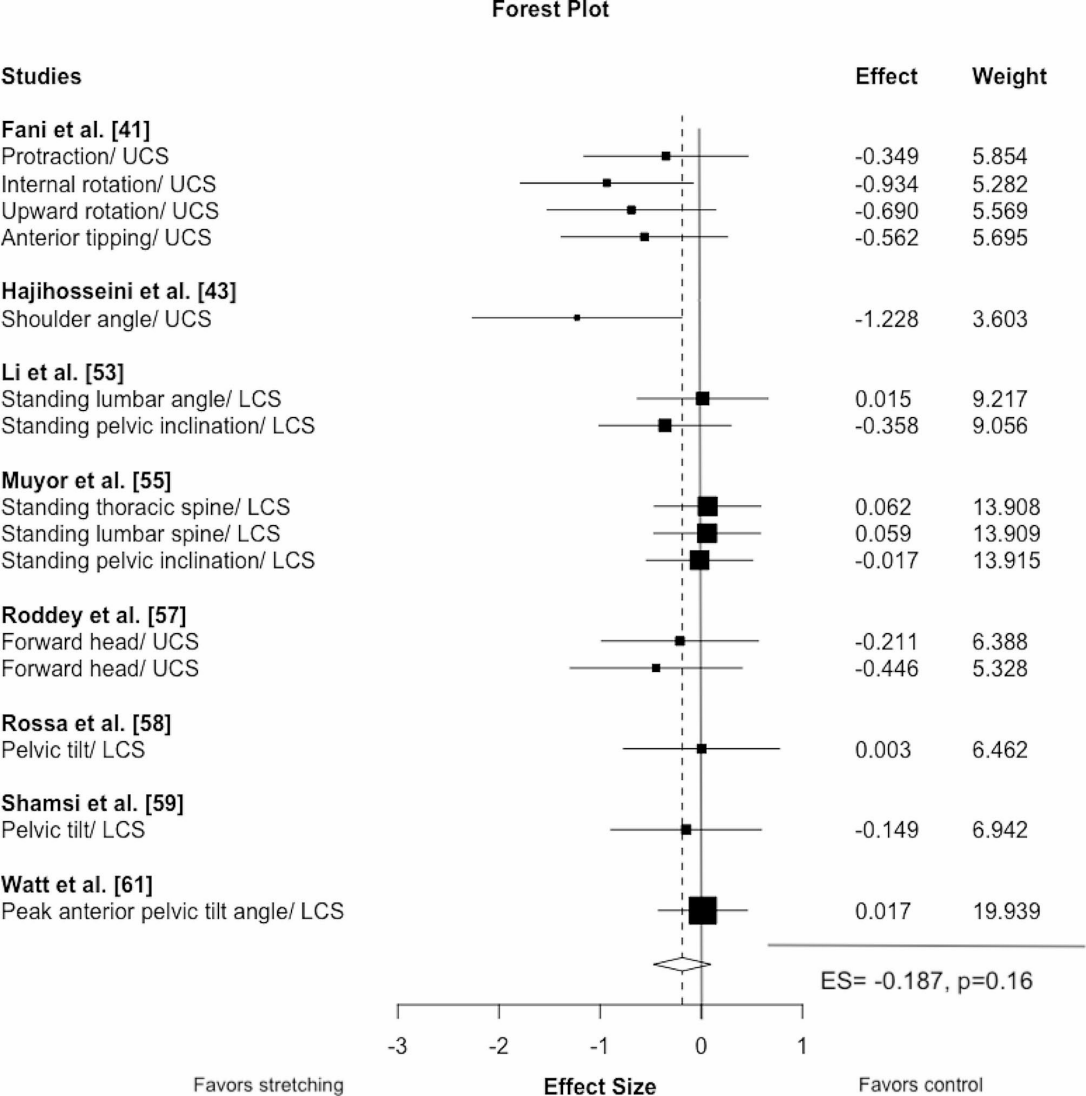
Forest plot for chronic stretching interventions on posture. Negative values illustrate effects favoring stretching compared to control. The effect size includes the 95% confidence interval

Neither acute stretching (d = 0.013, -3.33, 3.36 95% CI, *p* = 0.97, τ²=0.01, 2 studies, 3 ES) nor chronic stretching (ES=-0.19, 95%CI -0.47 to 0.1, *p* = 0.16, τ²=0.0, 8 studies, 15 ES) had an effect on posture. Likewise, subgroup analyses showed no impact of stretching in any of the tested body regions (pelvis/lumbar spine: ES=-0.04, 95% CI -0.17 to 0.09, *p* = 0.43, τ²=0.0, 5 studies, 7 ES; thoracic/cervical spine: ES=-0.44, 95% CI -1.03 to 0.16, *p* = 0.101, τ²=0.02, 4 studies, 8 ES; see Table 3; Fig. 2). The certainty about the evidence was moderate.

**Table 3:** Meta-analytic results providing effect size, 95% CI, significance and heterogeneity

Parameter	Effect size (95% CI)	*p*-value	Heterogeneity (τ²)
Acute stretching^a^	0.01 (-3.33 to 3.36)	0.97	0.01
Chronic stretching^a^	-0.19 (-0.47 to 0.10)	0.16	0.0
Chronic stretching (lumbar spine/pelvis)	-0.04 (-0.17 to 0.09)	0.43	0.0
Chronic stretching (thoracic/cervical)	-0.44 (-1.03 to 0.16)	0.10	0.02
Chronic strengthening^b^	-0.87 (-1.58 to -0.17)	0.02	0.4
Chronic strengthening (lumbar spine/pelvis)	-0.23 (-1.5 to 0.98)	0.25	0.0
Chronic strengthening (thoracic/cervical spine)	-1.04 (-1.69 to -0.40)	0.005	0.19
Chronic stretching^a^ vs. strengthening	0.81 (0.4 to 1.22)	0.004	0.02

^a^ negative values indicate beneficial impact of stretching on posture compared to the comparison group/control condition, ^b^ negative values indicate beneficial impact of strengthening on posture compared to the control condition, 95% CI = 95% confidence interval

#### Chronic Strengthening

**Fig. 3:**
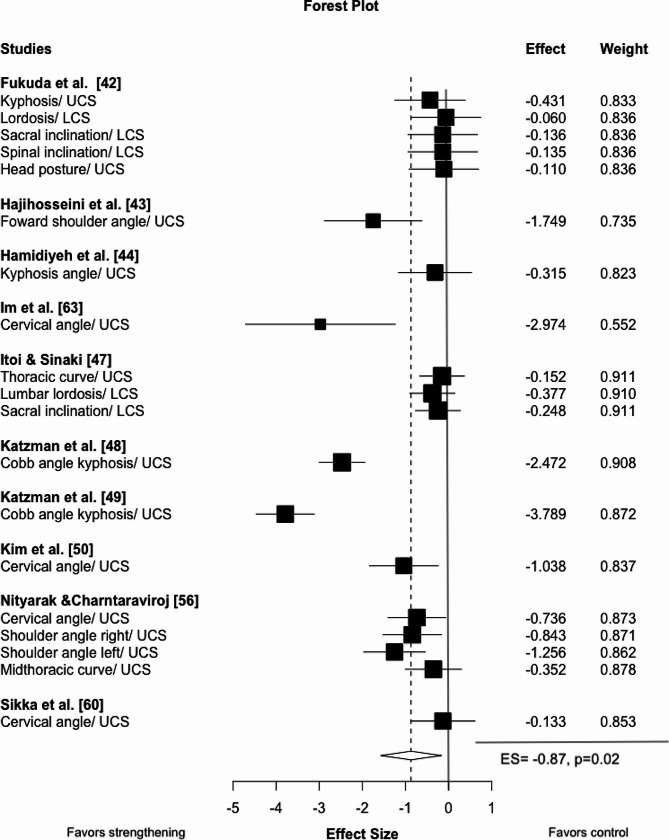
Forest plot for chronic strengthening interventions on posture. Negative values illustrate effects favoring strengthening compared to control. The effect size includes the 95% confidence interval

No study examined acute strengthening effects. Chronic strengthening had a large beneficial effect on posture (ES=-0.87, 95% CI -1.58 to -0.17, *p* = 0.02, τ²=0.4, 10 studies, 19 ES). According to the sub-analysis, no impact was identified in the pelvis and lumbar spine (ES=-0.23, 95% CI -1.45 to 0.98 *p* = 0.25, τ²=0.00, 2 studies, 5 ES), while a very large effect was found for the thoracic/cervical spine (ES=-1.04, 95% CI -1.69, -0.40, *p* = 0.005 τ²=0.19, 10 studies, 14 ES; Fig. 3). The certainty about the evidence was moderate.

#### Stretching vs. Strengthening

No study comparing acute stretch and strengthening interventions was found. For chronic interventions, a large effect in favour of strengthening exercise (d = 0.81, 0.4, 1.22 95% CI, *p* = 0.004, τ²=0.02, 6 studies, 9 ES) was detected. Since all studies but one focused on the thoracic/cervical spine region, no sub-analysis of body locations was possible.

## Discussion

Stretching of tight or shortened skeletal muscles represents one of the most popular strategies used to tackle muscle imbalance and postural impairments [26]. As early as 1997, Spring et al. [64] recommended it as the gold standard of posture treatment and twenty years later, the application of stretch was still described a viable method preventing hypertonia-induced muscular imbalance [65]. While recent reviews did not consider stretching as a stand-alone intervention [18, 66], Withers et al. [28] were only able to include one stretching study in their meta-analysis. Summarizing the effects of 12 chronic stretching studies, our systematic review is the first to extensively examine the foundation of this approach. Of note, in contrast to popular beliefs in practice, moderate-certainty evidence does not support the use of stretching when aiming to tackle imbalance-related posture deficits (e.g. hyperkyphosis or forward head posture). However, our analysis revealed a large effect of strengthening which also was superior in direct comparison to stretching. This finding confirms earlier speculations by Gonzalez-Galvez et al. [18] who reported combined stretching and strengthening to improve spinal posture, but suggested that only strengthening may be effective. As a consequence, exercise therapy for posture can be substantially economized by forgoing stretching tight muscles, and instead focusing on strengthening weakened muscles.

From a physiological point of view, it has been argued that chronic stretching of a tight or shortened muscle would lower its stiffness or tone. While stretching of two to eight minutes acutely reduced muscle stiffness [67–71], a rapid return to baseline occurred after a short recovery of only up to 20 min. This is highly plausible considering the mechanical role of the titin filament. The protein, which is attached to the myosin filament and the z-disk, has substantial elastic properties and after being lengthened (e.g., during a stretch), it helps to restore the original passive resting length. Acting as a molecular spring [72–74], it hence regulates the mechanical behavior of the muscle fiber [75]. Data collected in rabbits revealed that titin contributes up to 60% of the total passive stiffness of a skeletal muscle [76]. Experimentally disrupting the filament decreased passive tension by 50 to 100% [77]. Considering the elastic properties of titin and its role in passive muscle tension, the acute reductions in stiffness after stretching as well as the fast restoration of baseline values seem logical. Interestingly, the evidence of potential stiffness changes following chronic stretching treatments seems controversial. While in 2018, Freitas and colleagues [78] found stretch-mediated stiffness reduction in response to weekly volumes of up to 20 min over up to eight weeks unlikely, more recent literature found opposing results [79]. Yet, even if long-term stretching could reduce muscle stiffness, the causal relationship between decreasing stiffness of shortened muscles and improvements in posture remains speculative, calling for further exploration. While there is currently no evidence for positive chronic effects of stretching on posture, this might potentially be due to a lack of investigations that use sufficient stretching volumes meaning further research is necessary. Irrespective, it needs to be acknowledged that only two studies were available on acute stretch application. Additional research evaluating the immediate impact on posture is therefore warranted as well.

Besides reduced stiffness, another suggested effect of chronic stretching is an increase in muscle length. As such, one might expect the formation of new serial sarcomeres within the muscle-tendon-unit [80, 81]. Indeed, Williams and Goldspink et al. [82] observed a higher sarcomere number following long-term immobilization of animal limbs. However, on the one hand, immobilization cannot be readily compared to stretching and, on the other hand, the applicability of animal findings to humans is disputed [80]. Interestingly, titin does not only regulate the resting tension of the skeletal muscle but also appears to play an important role in structural adaptations. Van der Pjil et al. [83] described the importance of titin unfolding at high muscle lengths for sarcomerogenesis and with this, longitudinal (and parallel) hypertrophy. Even though viable, observations indicating a possible influence of chronic stretch training on structural properties were, to the best of our knowledge, exclusively made in animals [84, 85]. However, again, no transfer of longitudinal hypertrophy effects to humans was found [86]. Before 2020, stretch-induced chronic structural stretching adaptations were classified unlikely [78, 86], but within the past 5 years, evidence emerged that large stretching volumes (≥ 15 min per day, ≥6 weeks intervention period) have the potential to induce muscle hypertrophy, and with this, changes in tissue morphology [87, 88]. As, to date, no evidence could be found for longitudinal hypertrophy, it could be speculated that the studies matching the inclusion criteria of this systematic review did not perform stretching with the required stretching duration and/or intensity [87–89].

Contrarily to stretching, we found a large beneficial influence of strengthening on posture. However, the underlying mechanisms are a matter of debate. Surprisingly, there is a lack of conclusive research on resistance training-induced changes of the muscle’s passive mechanical properties [90]. In 1998, the hypothesis of increases in passive muscle stiffness as an adaptation to resistance training arose [91], leading to the recommendation to strengthen lengthened or weak muscle groups in muscle imbalance. The authors argued that hypertrophy would be associated with a larger number of parallel titin-myosin filaments, which, in agreement with the above-described evidence, would lead to a higher resting tension [91]. Indeed, in a ten-week strength training study, the authors reported a 30%-increase in passive tension without decreases in extensibility of the muscle. In another study, isometric resistance training led to an increase in core stiffness [92]. However, a recent systematic review found no stiffness changes in the long-term as a response to resistance training [93]. Of note, the review only included measurements with ultrasound elastography which allows assumptions on compressive tissue stiffness. Assuming specific resistance training adaptations occur following induction of tensile/shortening stress to the muscle, it seems necessary to distinguish between compressive and tensile or strain stiffness. Research on foam rolling effects revealed that decreases in compressive stiffness could be detected using elastography and indentometric methods, while this was not the case for tensile stiffness using passive resistive torque during stretch [94, 95]. As a consequence, it may be assumed that stiffness changes are specific to the applied stimulus (compression in foam rolling, but stretch-shortening in resistance exercise). Following this theory, it would still be possible that resistance training does only modify tensile stiffness, which would also align with the role of titin as a serial agent for passive tension regulation. In sum, more research is warranted in order to gain further insight into the mechanisms of strengthening-induced improvements of posture.

### Implications

Our findings have implications for clinical practice. As indicated, stretching is highly popular among therapists aiming to treat muscle imbalance and frequently recommended in the scientific literature [26, 64, 65]. Yet, the available evidence speaks strongly against this approach. In line with earlier speculations of Gonzalez-Galvez et al. [18], beneficial exercise effects seem rather attributable to strengthening, while stretching programs are ineffective. Consequently, when aiming to counteract muscular imbalances and to improve spinal and lumbopelvic posture, no evidence-based recommendation for the implementation of stretching can be given. Interestingly, we found a beneficial influence of strengthening for the thoracic and cervical spine region, while no changes were detected in the lumbar and pelvic region. On the one hand, effect sizes were in fact trivial to small for the lumbar spine and pelvis. On the other hand, with a total of only 5 ES from two studies, this region is under-researched. Future investigations, besides aiming to better understand the physiological adaptions of stretching and strengthening with regard to passive tissue properties (muscle, tendons, fascia) [90] and neuromuscular aspects [10], should be geared to provide more data on exercise treatments in the lumbar spine region.

## Conclusion

The common recommendation of stretching tight or shortened skeletal muscle to improve muscle imbalance and posture lacks scientific evidence (moderate certainty). In contrast, our review reinforces the role of strengthening weak antagonists which, however, was only effective in the thoracic and cervical but not in the lumbar spine (moderate certainty). Further well-designed RCTs, e.g. applying high stretch durations and experimental studies elaborating the underlying physiological mechanisms, are required to conclusively judge the role of treatments aiming to modify postural abnormalities.

### Electronic Supplementary Material

Below is the link to the electronic supplementary material.

Supplementary Material 1

## Data Availability

Data can be provided on reasonable request.

## Declarations

### Registration of the Study

The study was registered in the PROSPERO data base using the number CRD42023412854 and the title “Effects of stretching and strengthening exercise on spinal and lumbopelvic posture: a systematic review with meta-analysis”.

## Abbreviations

CI: Confidence interval
ES: Effect size
M: Mean
SD: Standard difference
SMD: Standardized mean difference.
