# Supplementary material for: Effects of Stretching or Strengthening Exercise on Spinal and Lumbopelvic Posture: A Systematic Review with Meta-Analysis
Source: Sports Med Open. 2024 Jun 5;10:65. doi: 10.1186/s40798-024-00733-5 (PMC11150224; doi:10.1186/s40798-024-00733-5)
Supplement: Supplementary file 1 — Supplementary Material 1 [file 40798_2024_733_MOESM1_ESM.docx]

**Supplemental Material**

**Appendix S1 PubMed search terms:**

(“spinal curv*” OR “spinal alignment” OR “spinal posture” OR “pelvic tilt” OR “forward head posture” OR FHP OR lordosis OR kyphosis OR “rounded shoulder” OR “protracted shoulder” OR “forward shoulder” OR “imbalance”) AND stretch*.


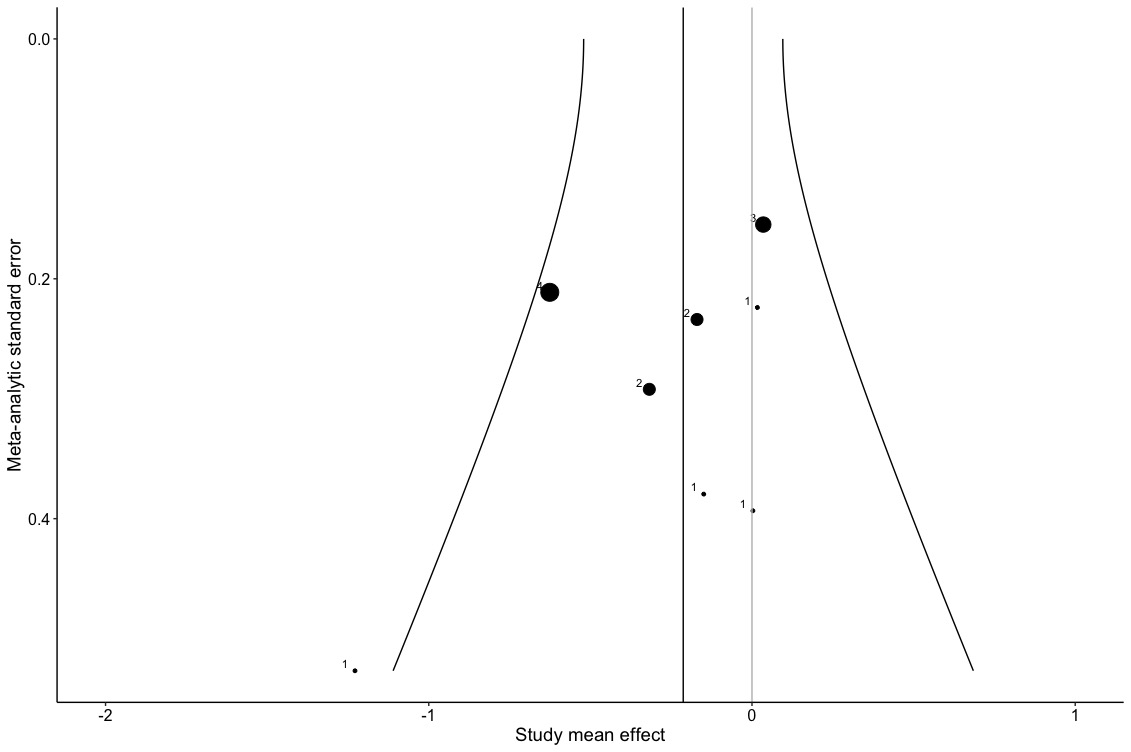


**Figure S1** illustrating the publication bias for chronic stretch studies


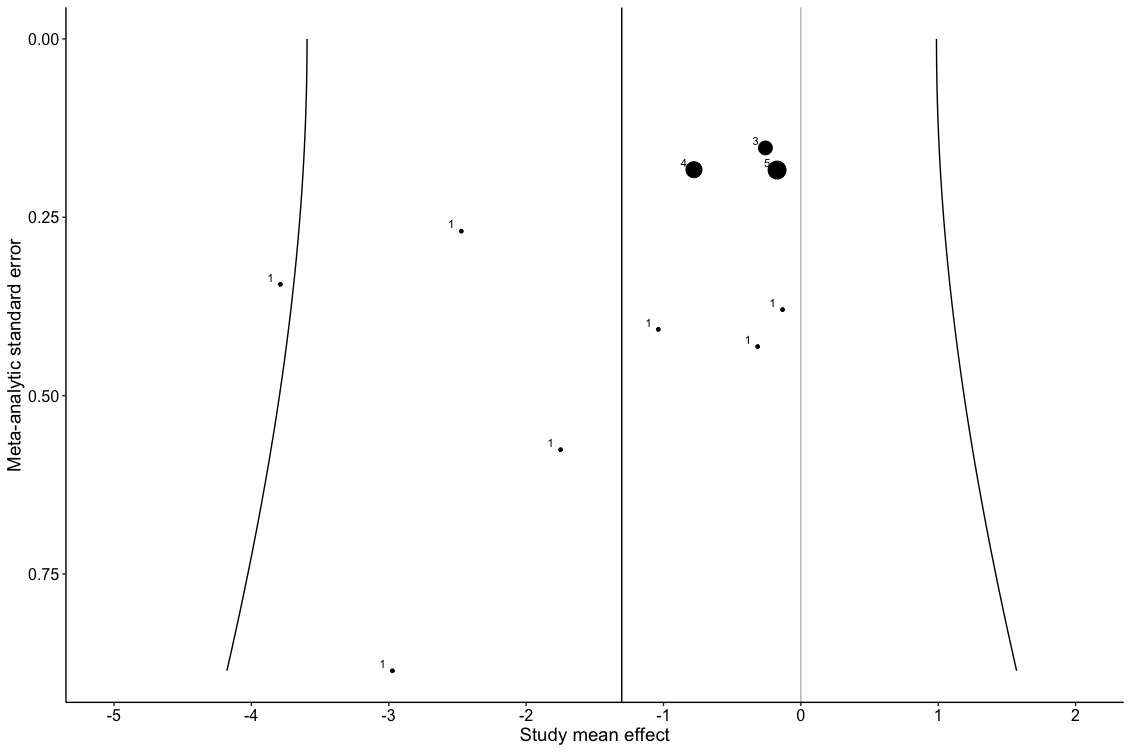


**Figure S2** illustrating the publication bias for chronic strengthening studies


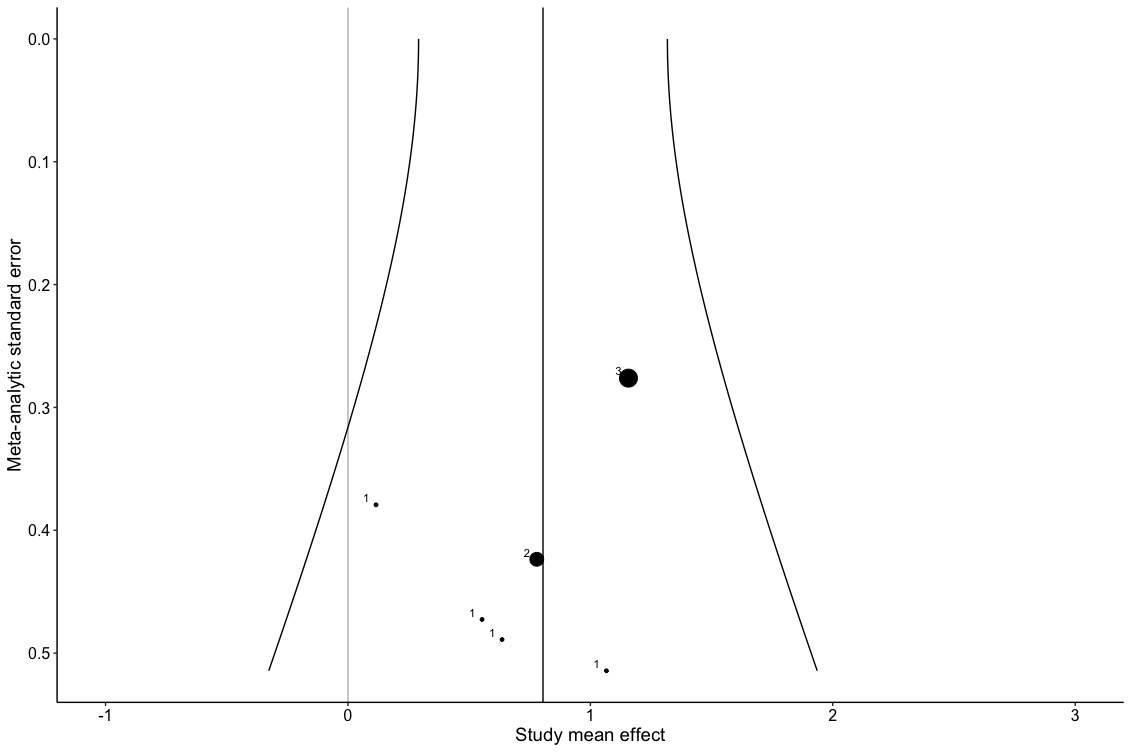


**Figure S3** illustrating the publication bias for chronic comparison between stretching and strengthening
